# Design of a Mobile App for Nutrition Education (TreC-LifeStyle) and Formative Evaluation With Families of Overweight Children

**DOI:** 10.2196/mhealth.7080

**Published:** 2017-04-13

**Authors:** Silvia Gabrielli, Marco Dianti, Rosa Maimone, Marta Betta, Lorena Filippi, Monica Ghezzi, Stefano Forti

**Affiliations:** ^1^ Fondazione Bruno Kessler High Impact Initiative on Health & Wellbeing Trento Italy; ^2^ Pediatra Libera Scelta Trento Italy

**Keywords:** mHealth, child, overweight, pediatrics, health behavior, evaluation studies

## Abstract

**Background:**

Nutrition and diet apps represent today a popular area of mobile health (mHealth), offering the possibility of delivering behavior change (BC) interventions for healthy eating and weight management in a scalable and cost-effective way. However, if commercial apps for pediatric weight management fail to retain users because of a lack of theoretical background and evidence-based content, mHealth apps that are more evidence-based are found less engaging and popular among consumers. Approaching the apps development process from a multidisciplinary and user-centered design (UCD) perspective is likely to help overcome these limitations, raising the chances for an easier adoption and integration of nutrition education apps within primary care interventions.

**Objective:**

The aim of this study was to describe the design and development of the TreC-LifeStyle nutrition education app and the results of a formative evaluation with families.

**Methods:**

The design of the nutrition education intervention was based on a multidisciplinary UCD approach, involving a team of BC experts, working with 2 nutritionists and 3 pediatricians from a primary care center. The app content was derived from evidence-based knowledge founded on the Food Pyramid and Mediterranean Diet guidelines used by pediatricians in primary care. A formative evaluation of the TreC-LifeStyle app involved 6 families of overweight children (aged 7-12 years) self-reporting daily food intake of children for 6 weeks and providing feedback on the user experience with the mHealth intervention. Analysis of the app’s usage patterns during the intervention and of participants’ feedback informed the refinement of the app design and a tuning of the nutrition education strategies to improve user engagement and compliance with the intervention.

**Results:**

Design sessions with the contribution of pediatricians and nutritionists helped define the nutrition education app and intervention, providing an effective human and virtual coaching approach to raise parents’ awareness about children’s eating behavior and lifestyle. The 6 families participating in the pilot study found the app usable and showed high compliance with the intervention over the 6 weeks, but analysis of their interaction and feedback showed the need for improving some of the app features related to the BC techniques “monitoring of the behavior” and “information provision.”

**Conclusions:**

The UCD and formative evaluation of TreC-LifeStyle show that nutrition education apps are feasible and acceptable solutions to support health promotion interventions in primary care.

## Introduction

### Background

Mobile technology has experienced impressive gains in popularity in recent years [1]. As people typically develop a strong attachment to their mobile devices and frequently keep this technology with them all the times, mobile platforms provide a critical source of information and motivation for engaging users with health interventions and behavior change (BC) [2-3]. Mobile devices increase the potential to promote healthy nutrition behaviors, and today nutrition and diet apps represent the fastest growing field of health promotion apps [4]. Mobile health (mHealth) apps for nutrition education can be suitable solutions to support parents’ involvement in childhood weight management interventions provided in primary care, as this is often the families’ first point of contact with the health care system [5,6]. The delivery of a primary care intervention by leveraging on digital and mobile support facilitates a higher compliance to it by the target users such as parents, who might have tight daily agendas, difficulties in tracking children’s weight-related behaviors over a day per week on paper diaries, and location difficulties [7]. Also, nutrition education apps make it possible to follow up healthy eating interventions long after their completion, a time when relapses are most likely to occur, thus supporting maintenance of healthy behavior in the long-term [8].

Today, a large consensus exists in recommending the development of mHealth interventions as based on evidence, BC theory and taxonomies, as well as on formative evaluation with the target user groups [9,10]. However, still a majority of children weight management apps do not use any recommended strategies or behavioral targets [11], and a few apps specifically target parents or families, which instead play a key role in supporting children’s adoption of healthy lifestyles [12,13]. Moreover, recent studies have shown that commercial mHealth apps are more engaging and better appreciated by users with respect to evidence-based mHealth apps [14], but the quality of information provided by commercial mHealth apps is often rated as quite poor [15,16]. This suggests that a thorough user-centered design (UCD) approach informed by theoretical-empirical knowledge, as well as by user engagement principles, is required today to ensure a wide adoption of the mHealth intervention provided [10,17-19].

Involving all relevant stakeholders and target users in the development of such interventions may help improve their effectiveness and acceptability [20,21]. Getting early feedback from end-user populations helps ensure quality and usefulness of content, and the involvement of stakeholders who play a role in providing care to or managing overweight children is the key to inform feasibility and usability of the solutions designed [22].

### User-Centered Approach

This study describes how a UCD approach [23] founded on primary care practices and requirements was deployed for developing the TreC-LifeStyle app for nutrition education, and how the validity of the mHealth intervention designed was tested with representatives of the target user group in a formative evaluation.

## Methods

### mHealth Intervention Design and Development

**Figure 1:**
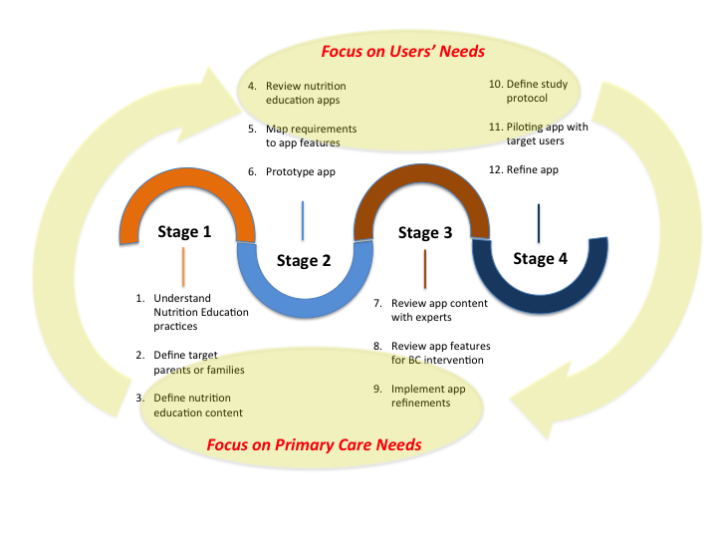
The design and development stages of the mHealth app intervention. BC: behavior change.

The design and development process of the TreC-LifeStyle mHealth intervention followed four main stages (Figure 1):

1. Collecting requirements from primary care professionals on nutrition education practices, guidelines, and on the typical profiles of parents or families that could most benefit from the intervention
2. Prototyping the app features based on the collected requirements and on a review of nutrition education apps already available in the market
3. Revising the prototyped features with pediatricians and nutritionists with respect to content and BC strategies
4. Deploying the intervention in a formative evaluation study with target families for further refinement of the mHealth app and intervention

In stages 1 and 3, several meetings were held in 2015 among the design team, involving experts in BC interventions, working together with 3 pediatricians and 2 nutritionists from primary care services in the Trento region (Italy). In stage 2, the design team reviewed state-of-the-art features in nutrition education apps available in the market, then mapped the requirements collected with the most appropriate BC techniques proposed by international standards and taxonomies [24]. It also developed a concept for providing the mHealth intervention in the most intuitive way, reducing as much as possible attention thefts by the app for children’s food intake reporting and monitoring by parents.

In stage 4, a protocol for the formative evaluation study was prepared by the design team and then deployed for understanding usability and feasibility of the mHealth intervention with target users.

### Nutrition Education Requirements

In stage 1, pediatricians helped better understand the nutrition education practices and typical interventions provided in pediatrics primary care to prevent children from being overweight [25], as well as the main obstacles met by parents and families in adopting healthy eating behaviors. A main objective for pediatricians is to teach parents how to adhere to the eating guidelines provided by the Mediterranean Diet and the Food Pyramid while educating their children on healthy nutrition and managing them. Parents of overweight or obese children aged 6-12 years often seek support from primary care services, they might be prepared to change, but often fail in putting into practice healthy eating practices. In recent years, pediatricians tend to recommend nutrition education programs based on Therapeutic Patient Education [26] rather than Dietetic Therapy [27], as prescriptive dietary approaches are often perceived as more stigmatizing of individuals’ behaviors and were found less acceptable and effective in achieving BC in the short- and long-term [28]. Educational approaches avoid an exclusive focus on children’s food intake calories control, but encourage parents to become more aware about their children’s lifestyle, including both eating behavior and physical exercise and the complex system of emotional perceptions related to food intake. Accurate, reliable, and personalized monitoring of children’s lifestyle is always a challenge during primary care intervention; mHealth apps can improve the delivery of these interventions, helping nutrition education to turn into effective BC, but they need to be enough motivating and engaging to be used by families to achieve the desired objectives.

### TreC-LifeStyle App Description

As an output of stage 2, a first prototype of the mHealth app was developed based on a mapping of the requirements collected from primary care experts with nutrition education features most likely to support the desired BC effectively (Table 1).

The mobile app is part of the TreC platform used in the Trento region as a citizen-controlled clinical record system allowing users to manage their own health and facilitating communications with health care professionals and institutions [29,30].

**Figure 2:**
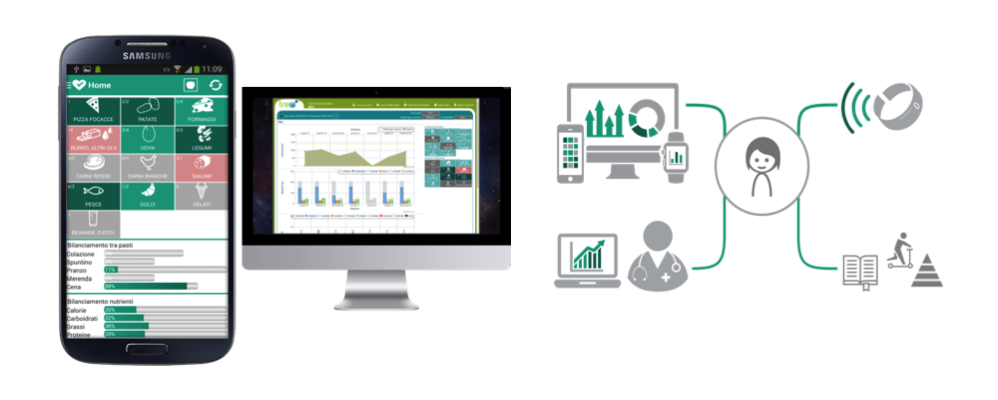
The TreC-LifeStyle mobile and Web platform.

In case of the TreC-LifeStyle solution, pediatricians can access data of their patients (child or parent) from a Web app dashboard (Figure 2) integrated into the TreC platform; they can configure the app intervention for each patient, prescribe it as a nutrition education intervention, and visualize data related to the intervention to inform follow-ups with patients. The TreC-LifeStyle prototype app was developed for Android devices only in this phase, with the plan of implementing both Android and iOS versions of the mobile app after the formative evaluation phase. To ensure confidentiality of the data acquired, the TreC-LifeStyle platform saves data in the local database of the user mobile phone device, in an anonymized format. The anonymized data are then transferred on to a server by means of rest calls requiring security authentication of level 2 (username and password). This way, the identity of the user-participant is only known by the pediatrician who has prescribed the mHealth intervention.

The target user of the TreC-LifeStyle intervention is the child-parent–dyad in need of overweight or obesity prevention support. In order to minimize possible acceptability concerns related to young children’s use (and possible abuse) of the mobile technology, the TreC-LifeStyle app is meant to be installed on the parent’s mobile phone and used primarily by the parent as a supporting tool for more precise monitoring of the child’s food intake and physical activity during the intervention. To ensure child engagement during the BC intervention, the child is asked to wear a bracelet (synchronized to the mobile app) for automatic tracking of daily steps to be reviewed with the parent on the TreC-LifeStyle home display and for checking the achievement of daily physical activity goals.

**Table 1:** Mapping of user requirements to TreC-LifeStyle app features and behavioral change techniques.

User requirements	App features	Behavioral change techniques
Time-saving, convenient, intuitive interaction	Food intake dashboard (including recommended portions and quantities), virtual coaching messages	Instruction on how to perform the behavior
Digital tracking of child’s food intake over time	Colors of food categories on dashboard, reports of meals and nutrient balance	Monitoring of the behavior
Tracking of compliance with healthy nutrition guidelines	Color or blinking of food boxes on dashboard, color of nutrient bars on reports	Feedback on the behavior, prompt or cues
Automatic tracking of child’s physical activity	Daily steps display	Review behavioral goal achievement
Motivation to increase physical activity	Steps label in gold	Rewarding
Memory aid for future food purchases	Shopping list	Information provision

**Figure 3:**
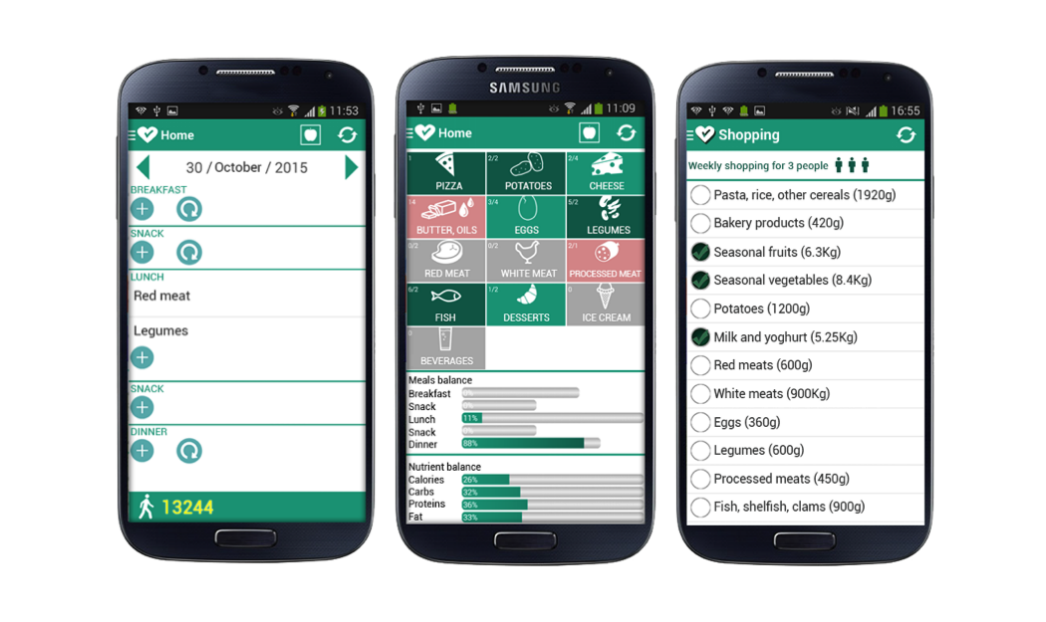
Screenshots of the TreC-LifeStyle app features: food-intake reporting, weekly report, and shopping list.

The concept design of TreC-LifeStyle app was based on the following principles:

1. The delivery of evidence-based nutrition education content, compliant with primary care practices, supporting knowledge and adoption of the Mediterranean Diet, Food Pyramid guidelines [31-33], and at least 10 K steps of daily physical activity by children.
2. A low-burden reporting of children’s food intake by parents by means of an intuitive food dashboard screen on the app (Figure 3), based on the food categories recommended by international and national guidelines on healthy nutrition [31-33], combined with monitoring, feedback, rewarding, and virtual coaching features proposed by international taxonomies of BC techniques [24].
3. Automatic tracking of children’s physical exercise by means of commercial devices like Jawbone and Misfit bracelets integrated with the app’s features: The number of daily steps detected from the wearable device is displayed on the home page of the mobile app, close to the display of the calories intake. When the child reaches the goal of 10 K steps per day, the steps label turns to gold to acknowledge the goal achievement.

Children’s daily food intake reporting is done by parents who play a key role in children’s nutrition management, by selecting food elements on the app dashboard with a few clicks (Figure 3): When correct food elements and portions are selected, the corresponding food box turns green, if food elements and portions exceed the recommended quantities, the box turns red whereas unselected food elements and portions remain gray. Gray boxes go on a blinking mode when the participants have filled in their daily food intake without including sufficient types and quantities of recommended food. This is to prompt the parent participant to align with the guidelines’ indications. Colored bars in the app display also help the parent participant to achieve a better balancing of nutrients in children’s meals; red bars are shown to indicate excessive quantities consumed in a meal or nutrient (eg, lipids, carbohydrates). The bars’ lengths are used to indicate at a glance that different meals have different importance during a day.

Additional monitoring and feedback features (Figure 3) allow the parent participant to (1) monitor the food consumed, in a day or week, (2) visualize calories intake distributed in the principal meals and nutrients through progress bars. Based on personal food intake statistics, the virtual coaching function sends to the user daily reports summarizing deviations versus correct nutrition behavior with respect to the healthy diet guidelines. The virtual coach also sends notifications reporting guidelines for healthy diet adoption during the first 11 days of intervention (1 per day), covering basic topics relevant to nutrition education. Other more detailed information about food properties are available and can be inspected by the user on the app.

The app also provides a simple shopping list feature, allowing a parent participant to mark food to be purchased over a week, with the possibility of changing the family size (Figure 3).

During stage 3, the prototype concept and design were presented to the clinical stakeholders involved in stage 1 who revised both the app content and the BC features.

A main suggestion provided by pediatricians was to keep the overall mHealth intervention within a duration period of 6 weeks, as this is aligned with the typical brief prevention programs delivered in primary care to families to support children’s weight management. Clinicians also advised to keep the food intake-reporting task on the app as simple and low demanding as possible for the participants in the initial phase of familiarization with the mHealth intervention. Based on these suggestions, a basic version of the app (vA) was provided to participants in the first 3 weeks of the intervention, not requiring an exact specification of children’s food intake, but a simple reporting of type of food and portions consumed by children. In the following 3 weeks of the intervention, the app automatically upgrades to a more advanced version (vB), where more precise quantities of food intake, dressing of food, and so on can be reported.

### Formative Evaluation

**Figure 4:**
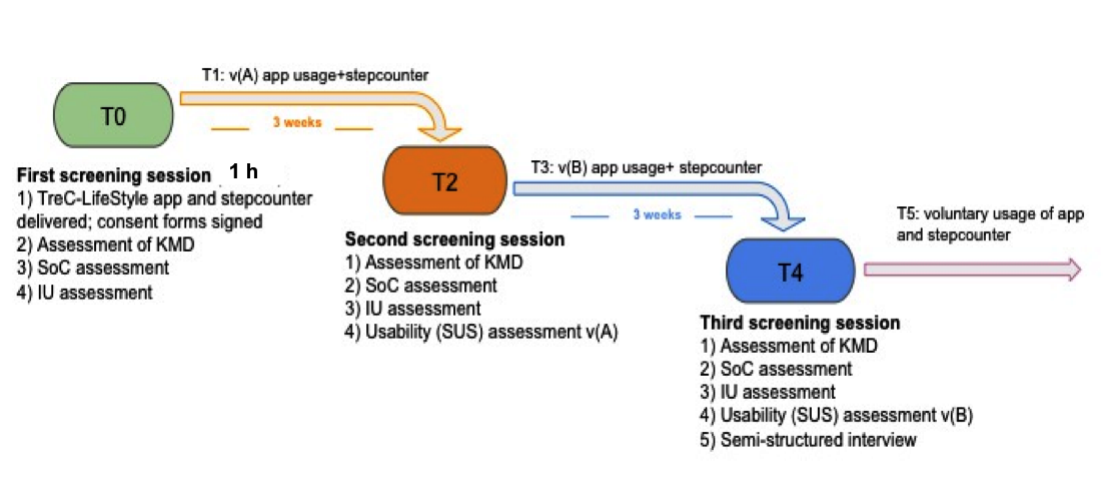
Protocol of the TreC-LifeStyle’s formative evaluation conducted in stage 4. vA: version A; vB: version B; KMD: knowledge of the Mediterranean diet; SoC: state of change; IU: intention to use; SUS: system usability scale.

In stage 4, a protocol for conducting a usability and feasibility study of TreC-LifeStyle was prepared and discussed with the clinical stakeholders to ensure alignment during the pilot. Researchers obtained ethics approval of the study protocol by their Institutional Review Board. As shown in Figure 4, the study involved 3 screening meetings with participants at the primary care offices. At the pilot start, to assess (1) parents’ knowledge of the Mediterranean Diet (KMD) guidelines; (2) state of change (SoC) for healthy nutrition-measured by an adapted version of the URICA-short-form scale, based on the 4 readiness to change states of the Transtheoretical Model of Change [34,35]; and (3) intention to use (IU) technology for nutrition education, as well as to train participants on usage of the app and of the activity tracker. After 3 weeks of the app vA usage to assess any change on the screening factors and evaluate parents’ perceived usability of the app (System Usability Scale Questionnaire [36]). After 6 weeks from the study start, to assess any change on the screening factors after using vB of the app and to investigate more in depth the participants’ experience with the mHealth intervention in a final semi-structured interview involving both parents and children. Participants were allowed to continue to use TreC-LifeStyle and the activity tracker for the following weeks, on a voluntary basis.

### Study Participants

A convenience sample of 6 families attending a pediatric center in the Trentino area for problems related to overweight children was recruited. The group included parents with children aged 7-12 years, classified as overweight (BMI 85th-94th percentile), familiar with the use of mobile phone technology. Families with children already affected by obesity and by motor impairments limiting physical activity were excluded.

Participants were contacted and invited to join the study by phone. If they were interested in participating, an appointment for a recruitment and introduction session was made at the pediatric center. Parent participants provided written informed consent prior to their inclusion in the study.

### Data Analysis

Descriptive frequency analyses were conducted on the quantitative data about the use of the TreC-LifeStyle app with respect to the prescribed intervention phases. At the qualitative level, positive, negative, and improvable aspects of the participants’ experiences with the TreC-LifeStyle app versions were descriptively coded. Interviews were transcribed verbatim and participants were assigned a numerical indicator (eg, Parent 1=P1). Transcribed data were subjected to an inductive thematic analysis procedure [37], which enabled the researchers to break down the data and identify some core themes that could have relevant implication for the future refinement of the mHealth intervention. Three team members completed the analysis independently. Transcripts were read several times to identify units of meaning in the data. Units of data (quotes) were isolated and similar quotes were grouped together as themes. Data coded within each theme were compared to ensure exclusivity. Themes were placed into 1 of 3 inductively generated groups according to positive feedback, negative feedback, and recommendations for improvements expressed by participants’ quotes. These groups were initially created by 1 researcher and then independently verified by 2 other researchers. Themes arising from postintervention interviews were compiled and presented in a tabular form. A meeting among the 3 researchers was finally held to revise the list of themes in the groups and resolve any difference.

## Results

### Screening Outcomes

The participants group included 6 parents (5 females, 1 male) of 6 overweight children (2 females, 4 males), mean age 9.16 years (SD 2.13). No participant dropout occurred during the prescribed intervention phase (first 6 weeks of app usage).

Parents’ KMD was good before the intervention and slightly improved at the end of the intervention (mean score from 12.5 to 12.70 on a total score of 15) with more than 80% of correct responses provided.

It was found that 4 out of 6 parent participants were classified at contemplation SoC before the intervention, but moved to action level after the 6 weeks’ intervention (Table 2).

All parent participants expressed a strong IU mobile apps for supporting the acquisition of healthy nutrition and lifestyles by children, both before and after the intervention (average rating was 5 on a 5-point Likert scale).

### Usage Patterns and Usability of the TreC-LifeStyle App

During the pilot study, TreC-LifeStyle’s app was used by both parents and children in 3 out of the 6 families involved (P2-child aged 10 years, P3-child aged 12 years, and P6-child aged 11 years). In the above three cases, both children and parents shared the food-intake reporting task and used the app during the study. In other cases, it was the parent participant using the app for food-intake reporting and analyzing feedback, whereas the child wore the activity tracker and checked with the parent the achievement of the activity goal of 10 K steps per day on the app mainly.

The adherence to the mHealth intervention was very high during the first 6 weeks, with a percentage of meals inserted always higher than 90% (mean number of meals inserted per day 2.79), showing that participants kept track of most of their meals and were compliant with the intervention request. This finding also shows a good user acceptance and effectiveness of the BC techniques “instruction on how to perform the behavior,” “feedback on the behavior,” and “prompt or cues” implemented by means of the food intake dashboard of the app and of the virtual coaching function. In the follow-up phase, when families could use the app on a voluntary basis, 2 participants almost dropped out (P2 and P6) and the other participants lowered interaction with the app considerably (mean number of meals inserted per day 0.44). The follow-up phase occurred during children’s summer holidays, and this had a strong impact on families’ daily routines and usage patterns (eg, in summer schools children were not allowed to use wearable devices for security reasons).

The frequency of usage of the daily statistics on food intake reported (Table 2), as well as of weekly statistics and of the shopping list feature shows that these app’s functionalities were rarely used by parent participants during the study. Therefore, a refinement of the app features supporting the BC techniques “monitoring of the behavior” and “information provision” is required, in order to make these features more engaging and useful from a user’s perspective.

**Table 2:** State of change (SoC), pre-post intervention, daily statistics visualization, percentage of goal achievement (10 K steps per day) in the last 3 weeks of intervention, difference of total deviations from healthy diet in week 1 and week 6 by participants (n=6).

Participant	SoC pre-post intervention	Daily statistics visualization	% of physical activity goal achievement–last 3 weeks of intervention	Difference of total deviations from healthy diet in week 1 and week 6
P1	Maintenance	62	121	0
P2	Contemplation-action	9	133	2
P3	Contemplation-action	9	74	0
P4	Contemplation-action	195	138	1
P5	Maintenance	18	101	−1
P6	Contemplation-action	0	113	−5

The result of children’s physical activity tracking was very positive, with most of the children participants reaching, and even overpassing the daily goal of 10 K steps (Table 2). Therefore, we can state that the app features related to the BC techniques “review behavioral goal achievement” and “rewarding” contributed well in supporting children’s engagement with the TreC-LifeStyle intervention and their motivation to reach physical activity goals.

By comparing the number of food portion deviations from healthy diet guidelines during week 1 (W1) and week 6 (W6) of the study (beginning and ending weeks of the intervention), we observed that 2 children showed stable behavior, 2 children made a few more deviations, and 2 children made fewer deviations (Table 2). It was noted that the absolute number of deviations per week was not very high in both weeks, which indicates a persisting commitment of participants to comply with the objectives and indications provided by the nutrition education intervention.

Overall, parent participants rated the usability of TreC-LifeStyle app very positively. Both app versions vA and vB were assessed as being at the *best imaginable* level of usability, obtaining SUS mean scores of 95 and 97.92, respectively.

### Qualitative Evaluation of the Intervention

Table 3 presents a list of core themes regarding positive, negative, and improvable aspects of the app design reported by participants during the interviews carried out at the end of the mHealth intervention.

**Table 3:** List of core themes and relative example quotes derived from the qualitative analysis of participants’ post-intervention interviews.

Themes groups	Themes	Example quote
Positive feedback	Simplicity of food-intake reporting	*I prefer vB as it is more precise regarding calories...* *and food quantities* *...* [Parent, P6]
	Conscious food purchases	*We have changed our habits at the store...* *we do not care much about special offers anymore.* [Parent, P3]
	Family awareness of dietary choices	*I realized that I used to prepare French fries or fried food too often...* [Parent, P6]
	Influence on meals preparation	*Mom and daddy checked the app to decide what to prepare for lunch and dinner...* [Child, P3]
	Children’s goal-driven motivation	*I use the app more if there is the bracelet...* *I want to achieve the goal 100%* *...* [Child, P2]
Negative feedback	Difficulties with food portion assessment	*It was not easy sometimes to know the portion of food consumed...* [Parent, P5]
	Poor engagement with secondary app’s features	*We did not understand much the functionality “shopping list” so we never used it* [Parent and child, P1]
	Problems with children’s use of wearable trackers	*I could not wear the bracelet during some of the sport activities at school, it was forbidden* [Child, P2]
Recommendations for improvement	Visual display of calories intake or burnt	*It would be interesting to have the app showing information about burned calories or intake* [Parent, P4]
	Support to food categorization	*It would be good to get some app’s help on categorization of some particular foods, like “polenta”* [Parent, P6]
	Healthy recipes provision	*I would like to receive suggestions on recipes for next meals, as some days it is not easy to find new ideas for meals preparation* [Parent, P5]

All interviewees reported that both the app versions were intuitive to use and valuable as educational tools to deepen their knowledge of healthy nutrition and of the Mediterranean Diet. Most participants expressed a preference for vB over vA, by saying that vA was good enough to get some general knowledge of healthy nutrition, but vB was more precise and allowed a more accurate input of food intake also in terms of quantity.

Some participants also thanked the app intervention, as their family had become more aware about dietary choices over a week, and less influenced by special offers at the store when buying food.

Participants reported being influenced by the feedback provided by the mobile app, which marked the categories of food in red when deviating from a healthy diet. The food categories marked in red were checked every evening, after dinner, affecting decisions about meals to be prepared the day after.

A strong support for motivating the app usage over the intervention was provided by the associated wearable devices used to track the physical activity of children, which triggered participants’ interest in monitoring and reflecting over calories’ intake versus consumption during the day. Parents reported that children were very interested in monitoring their activity levels and they tried to comply with the 10 K steps per day goal every day, as confirmed also by the activity logs.

Participants found some difficulties in reporting food intake especially with vA, as they could not find out how to specify, approximate, or exact food quantities, as well as the dressing of the food consumed.

Most participants did not use at all or accessed a few times the weekly reports of the monitoring feature and the shopping list feature. It is likely that participants did not find these features particularly useful, which turned out not to contribute much to the user engagement with the app.

Some participants also reported issues with the use of the Jawbone or Misfit devices to be worn by their children during the intervention. For example, during sport activities, children were not allowed to wear the device and this prevented a full synchronization of the physical activity tracking with the diet monitoring features.

Participants were recommended to refine the mobile app by including a visual display of balance between calories intake or burning, which was not provided in the tested app versions. They also asked to facilitate as much as possible the input of some types of food that were less obvious to categorize (eg, corn item under cereals). Moreover, they proposed adding app suggestions on healthy recipes for next meals preparation, in order to consolidate their adoption of the Mediterranean diet in their daily lives.

## Discussion

### Principal Findings

To our knowledge, this is one of the first studies documenting the UCD of an mHealth app intervention for nutrition education targeting children aged 7-12 years, developed starting from the needs and practices of primary care in pediatrics, also investigating in depth the user compliance and experience with the intervention by 6 families over a period of 6 weeks.

All participants used the app regularly during the intervention period, showing a good compliance of users with the nutrition guidelines and the physical activity goals. Shared usage of the mobile app was observed especially in the families of children aged 10-12 years, so this might further suggest the acceptability and appropriateness of mHealth solutions for this target user group. As this was a pilot study aimed at testing and further improving our mobile app before its larger deployment, we focused on the usability and feasibility of the mHealth intervention, aimed at realizing an effective human and virtual coaching system for nutrition education programs in primary care.

### Design of an mHealth Intervention for Obesity Prevention in Pediatric Primary Care

The main difference between our TreC-LifeStyle app and commercial nutrition apps is that it targets families of overweight children attending pediatric primary care programs. Therefore, it is fully integrated with health promotion knowledge and approaches based on clinical evidence and it allows tailoring of the intervention to the specific needs and profile of each participant (eg, configuration of the app based on children’s age and weight by the pediatrician before prescription). Beyond supporting a more precise assessment and personalized intervention for obesity prevention in childhood, the TreC-LifeStyle approach has the potential to scale-up to become a useful tool for delivering prevention programs to the target population groups, by leveraging on participants’ motivation, knowledge, and self-management skills [38] combined with clinical coaching and support by pediatricians. From a health care perspective, the TreC-LifeStyle platform is an effective cost-benefit solution for collecting data on children’s behaviors and lifestyle more reliably, which can be used during primary care visits to inform doctor-patient dialog and facilitate BC goals [39]. This is also aligned with previous research, showing that stand-alone mHealth technologies are unlikely to drive BC, but are most useful when used concurrently with traditional care [40,41].

### User Experience of the Mobile App

The analysis of the mobile app usage by participants showed a very high adherence to the intervention over its 6 weeks’ duration, with a mean number of meals inserted per day of 2.79. The most frequently used features of the app were the food intake reporting and feedback dashboard, as well as the monitoring of the physical activity levels, that turned out to be also the most appreciated functionalities of the app from the participants’ subjective reports. These results are consistent with previous research, which shows the benefit of mHealth apps in supporting participants’ self-regulation [42] and an increasing of physical activity [43,44].

However, we observed a considerable drop in the number of daily meals inserted in the app during the follow-up period (mean 0.44), which confirms how food intake recording might impose a high level of burden on users and represent a main challenge if requested out of BC interventions provided by health care providers [45]. A revised version of our mobile app will feature 3 different steps or levels of nutrition education to go through, raising first the user’s awareness on categories of food consumed over a week, then on portions, and finally on balance of nutrients. This way the educational support provided by the app and virtual coaching system developed is expected to help the user to deepen their learning of healthy eating behaviors, step by step, unlocking contents at growing levels of detail.

Usability of TreC-LifeStyle was rated as very good by all participants. A positive effect of the intervention was also observed in terms of parent participants’ SoC, achieving action or maintenance levels for all participants after the first 3 weeks of the intervention. However, a deeper analysis of participants’ comments on their user experience with the app indicated that additional support in defining or inserting food quantities should be provided. Another requested improvement of the app regarded the visual display of the ratio between calories intake and burnt and a possible replacement of the shopping list feature with a more engaging support in recommending recipes preparation compliant with healthy dietary guidelines. It is likely that the design of some app features related to the BC techniques “monitoring of the behavior” and “information provision” was not optimal for ensuring parents’ engagement, which is important for BC [46]. In our revised app, the virtual coaching function proactively invites the user to consult the reporting features periodically (eg, in the evening, weekend) and takes into account user’s current deviations from healthy guidelines to propose goal settings and suggestions relevant to rebalancing food intake and unhealthy behaviors.

### Limitations

There are several limitations in this study. First, developing an mHealth intervention by deploying UCD methodology is rather time- and resource-intensive [9-47]. Some refinements of the features developed need to be implemented before a future large-scale deployment of our app, and going through the different phases of our intervention design took much longer than the typical design time frame of commercial apps for health promotion [48]. Another limitation is that our pilot study involved a small group of participants (parent participants were mainly mothers), which may not reflect the need of other families of overweight children in our target population.

However, to our knowledge, this study is one of the first to assess the usability and feasibility of a mHealth app intervention targeting families of overweight children aged 7-12 years over a 6-weeks' deployment and follow-up period in a primary care setting. Relevant mHealth solutions and evaluation studies are reported in [49,50], but they were targeting families of children or adolescents aged 10-17 years. All our participants used the app frequently over the intervention weeks, reporting positive effects in terms of children compliance with nutrition and physical activity goals.

TreC-LifeStyle mobile app has the potential of better supporting primary care clinicians in providing effective prevention and health promotion programs related to children’s lifestyles, but further improvements of the app in terms of long-term user engagement, nutrition coaching, and food-intake monitoring functions need to be implemented before a next large-scale trial deployment.

### Conclusions

TreC-LifeStyle mHealth intervention has the potential to become an effective solution for supporting health promotion and prevention programs for children’s weight management in primary care. The UCD and clinically founded methodology used to design the app—which combines healthy lifestyle guidelines, food-intake monitoring and intuitive feedback, as well as Web-based data sharing with pediatricians for evidence-based dialog—is a significant advance over the functionality of current commercially available food journaling apps, and may represent the kind of mHealth interventions best fitting the requirements of primary care practice. Future refinements of the app ensuring user engagement and maintenance of healthy behaviors after the intervention are to be considered and tested further.

## Abbreviations

BC: behavior change
eHealth: electronic health
IU: intention to use
mHealth: mobile health
SD: standard deviation
SoC: state of change
SUS: system usability scale
UCD: user-centered design
vA: version A
vB: version B
